# Reconstruction techniques for accelerating dynamic cardiovascular magnetic resonance imaging

**DOI:** 10.1016/j.jocmr.2025.101873

**Published:** 2025-03-06

**Authors:** Andrew Phair, René M. Botnar, Claudia Prieto

**Affiliations:** a School of Biomedical Engineering and Imaging Sciences, King’s College London, London, United Kingdom; b Escuela de Ingeniería, Pontifica Universidad Católica de Chile, Santiago, Chile; c Millennium Institute for Intelligent Healthcare Engineering, Santiago, Chile; d Instituto de Ingeniería Biológica y Médica, Pontifica Universidad Católica de Chile, Santiago, Chile; e Technical University of Munich, Institute of Advanced Study, Munich, Germany

**Keywords:** Dynamic cardiac MRI, MRI cine, MRI reconstruction, Parallel imaging, Spatio-temporal redundancy, Compressed sensing

## Abstract

Achieving sufficient spatial and temporal resolution for dynamic applications in cardiovascular magnetic resonance (CMR) imaging is a challenging task due to the inherently slow nature of CMR. In order to accelerate scans and allow improved resolution, much research over the past three decades has been aimed at developing innovative reconstruction methods that can yield high-quality images from reduced amounts of k-space data. In this review, we describe the evolution of these reconstruction techniques, with a particular focus on those advances that have shifted the dynamic reconstruction paradigm as it relates to CMR. This review discusses and explains the fundamental ideas behind the success of modern reconstruction algorithms, including parallel imaging, spatio-temporal redundancies, compressed sensing, low-rank methods and machine learning.

## Graphical abstract

**Figure:**
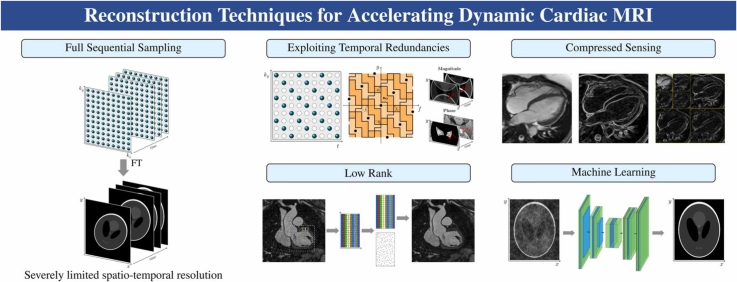

## 1 Introduction

The primary challenge presented by dynamic cardiovascular magnetic resonance (CMR) imaging is speed; is magnetic resonance imaging (MRI) a fundamentally slow imaging modality, but to be of clinical use dynamic magnetic resonance (MR) images must be acquired with sufficient spatial and temporal resolution. The acceleration of CMR scans, particularly in dynamic imaging applications, is therefore crucial to the success of modern CMR. This has been achieved in three key ways: hardware development, advanced sequence design, and by reducing the amount of data acquired during a scan. It is the third of these approaches that forms the focus of this review, for, as we shall see, when the amount of acquired data is reduced aliasing and undersampling artifacts are introduced into MR images unless sophisticated image reconstruction techniques are applied.

Reconstructing good-quality images from undersampled data sets is a complicated problem, and has been the focus of much research over more than 30 years. By exploiting spatial and temporal correlations in the data, additional information provided by multi-coil arrays, known prior information about the tissues being scanned or the nature of medical images, estimated motion fields and, most recently, machine learning, image reconstruction algorithms have been devised that continue to push the boundaries of what is possible in dynamic MRI.

In this review, we aim to provide the reader with an understanding of the concepts and ideas that underlie modern reconstruction algorithms. The amount of published literature on this topic is vast. As such, we focus on those particularly prominent methods which have either been incorporated into routine clinical practice or have introduced ideas fundamental to the further development of reconstruction techniques.

The review is organized as follows. In Section 2, the relationship between an MR image and the acquired data is explained and the source of undersampling artifacts is explored. In Section 3, some of the early techniques that were proposed for dynamic image reconstruction are discussed. Section 4 focuses on parallel imaging, and the seminal parallel imaging methods SENSE (sensitivity encoding) and GRAPPA (generalized autocalibrating partially parallel acquisitions). Although these are not specific to dynamic imaging, they are pivotal to many dynamic-specific methods proposed since. Section 5 introduces methods that utilize high-dimensional sampling theory and explicitly exploit spatio-temporal redundancies. In Section 6, iterative reconstructions regularized by sparsity constraints (compressed sensing [CS]) or low-rank assumptions are discussed. In Section 7, the emerging wave of techniques based on deep neural networks is discussed. Finally, some concluding remarks are provided in Section 8. A summary of key methods covered is included in Table 1.

**Table 1:** Summary of key selected reconstruction approaches discussed in this review.

Method	Acceleration strategy	Details
Full sequential sampling	None	Each frame acquired as a fully sampled image.
Key-hole	Temporal averaging	Initial acquisition of outer k-space reused throughout scan.
Restricted FOV	Temporal averaging	Motion limited to small image-space FOV.
SENSE	Parallel imaging	Image-space unaliasing algorithm. Requires coil profiles. Auto-calibrating and non-Cartesian variants.
GRAPPA	Parallel imaging	k-Space synthesis algorithm. Auto-calibrated with ACS. Also non-Cartesian variants.
SPIRiT	Parallel imaging	Self-consistent GRAPPA-like k-space synthesis for arbitrary trajectories. Auto-calibrated with ACS.
UNFOLD	Temporal filtering	Staggered k-space sampling induces aliasing, resolved through temporal frequency filtering.
k-t BLAST/k-t SENSE	Spatio-temporal	SENSE-like unfolding algorithm in high-dimensional x-f domain. Single coil/multi-coil.
k-t GRAPPA	Spatio-temporal	GRAPPA-like k-space synthesis in high-dimensional k-t domain.
k-t SPARSE/k-t SPARSE-SENSE	Compressed sensing/CS and parallel imaging	Sparsity imposed in wavelet domain (spatial) and Fourier domain (temporal). Incoherent artifacts from random sampling. Single coil/multi-coil.
k-t FOCUSS/MASTeR	Compressed sensing and motion correction	Sparsity imposed between separate, motion-corrected temporal frames.
XD-GRASP/5D Free-running	Compressed sensing and multiple temporal dimensions	Sparsity imposed along multiple temporal dimensions (e.g. cardiac and respiratory).
k-t SLR/k-t PCA	Temporal low-rank	Low-rank approximation imposed on pixel time series/temporal frequency profiles.
L+S	Compressed sensing and low-rank	Models dynamic image as slowly varying low-rank component and sparse dynamic component.
Image-/k-space- to-image networks	Deep learning	Deep neural network learns correlations between undersampled image/k-space and fully sampled image.
Unrolled networks	Deep learning	Iterative reconstruction alternating between denoising network and data consistency.
SSDU	Deep learning	Splits training data for network input and loss calculation. No fully sampled training data required.
Subject-specific networks	Deep learning	Network trained specifically for every scan.

*FOV* field-of-view, *SENSE* sensitivity encoding, *GRAPPA* generalized autocalibrating partially parallel acquisitions, *SPIRiT* iterative self-consistent parallel imaging reconstruction, *ACS* auto-calibration signal, *UNFOLD* unaliasing by Fourier-encoding the overlaps using the temporal dimension, *CS* compressed sensing, *MASTeR* motion-adaptive spatio-temporal regularization, *XD-GRASP* extra-dimensional iGRASP, *5D* five-dimensional, *SLR* sparsity and low-rank, *PCA* principal component analysis, *SSDU* self-supervised via data undersampling, *iGRASP* iterative golden-angle radial sparse parallel MRI, *MRI* magnetic resonance imaging, *FOCUSS* focal underdetermined system solver

## 2 Image reconstruction in MRI

During an MRI scan, a radio-frequency (RF) pulse irradiates protons within a slice or volume and causes them to transition to an excited, energetic state. Following the pulse, as the protons relax back to their ground state, they re-emit the energy as RF radiation. It is the strength of this radiation that is measured by the receive RF coils in the scanner, and these measurements form the MRI data set. However, a coil cannot distinguish where the radiation it measures originates from. Rather, it measures only the combined strength of all the radiation, across the entire imaging volume.

The innovation that made image reconstruction possible in MRI was the use of gradient coils, switched on and off throughout the scan, to Fourier-encode the acquired data. Each data sample still contains information about the entire image, but that information now corresponds to the image modified by a spatial encoding pattern which is induced by the cumulative effect of the gradient coils up to the time at which the sample is acquired. Specifically, the sample records the extent to which a given spatial frequency is present in the image. Large objects that vary smoothly and slowly across the image have low spatial frequency, whereas image texture, with no large-scale structure and rapid variation across the image, has high spatial frequency.

When a Fourier-encoding acquisition scheme is applied, the acquired data samples lie in the Fourier domain, known as k-space in MRI. In two dimensions, k-space is a coordinate system constructed from the *k*_*x*_ and *k*_*y*_ variables, which correspond to spatial frequency in the *x*- and *y*-dimensions of the image, respectively.

Depending on the acquisition, an additional third k-space dimension, *k*_*z*_, may also be considered, corresponding to the third dimension, *z*, of a three-dimensional (3D) image. Acquiring data in this manner enables the image to be recovered via an inverse Fourier transform, as described in Appendix A.1.

In practice, only a finite number of discrete k-space samples can be obtained, and we only wish to represent the image with a finite number of pixels or voxels (image-space samples). Hence, the discrete inverse Fourier transform is instead utilized. This also allows fast image reconstruction, since for Cartesian sampling it can be implemented very efficiently using the fast Fourier transform (FFT) algorithm [1].

The effect of the discretization can be understood via the convolution theorem, which states that the multiplication of two functions in k-space is equivalent to the image-space convolution of the inverse Fourier transforms of those functions. We consider the discretization to be a multiplication by the sampling function $A\left(k_{x},k_{y}\right)$, which consists of a Dirac delta function centered at each sample location and has zero value elsewhere. The inverse Fourier transform of $A\left(k_{x},k_{y}\right)$ is called a point-spread function (PSF), since it will be convolved with the image and in doing so spreads each pixel of the image.

**Fig. 1:**
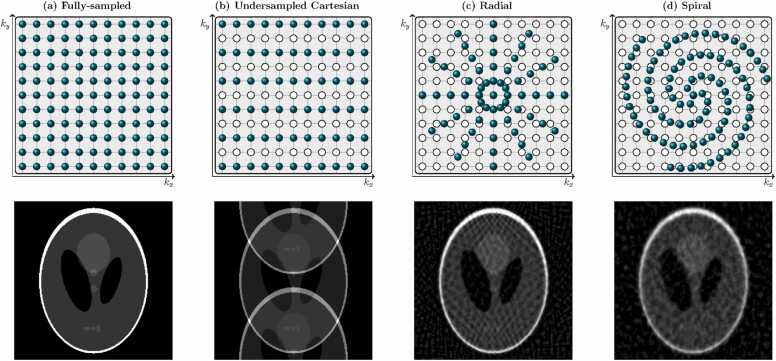
k-Space sampling trajectories (top row) and corresponding reconstructions of the Shepp-Logan phantom (bottom row) for (a) a fully sampled Cartesian acquisition, (b) a two-fold undersampled Cartesian acquisition, (c) an undersampled radial acquisition, and (d) an undersampled spiral acquisition. In the k-space diagrams, white circles represent unacquired Cartesian samples and green circles represent acquired samples

For k-space sampling on a regular Cartesian grid, the PSF consists of isolated individual points on a regular grid extending infinitely in all image dimensions, with their separation inversely proportional to the spacing between k-space samples. This means each pixel value is replicated an infinite number of times and the overall effect is a tessellation of the image. When the k-space samples *b*_*n*,*m*_ are acquired on a Cartesian grid with spacing consistent with the Nyquist-Shannon sampling theorem [2], [3], the separation between replicated pixels is equal to the image field-of-view (FOV), there is no overlap of image tessellations and the discrete inverse Fourier transform allows the image pixel values *ρ*_*u*,*v*_ to be reliably recovered from the k-space data. This is illustrated in Fig. 1a where a Cartesian sampling pattern in k-space returns an example image, the Shepp-Logan phantom, perfectly.

However, when k-space samples are acquired only on a subset of the Cartesian grid points, or on a non-Cartesian trajectory of k-space locations that lie between the grid points, the Nyquist criterion may be breached and image aliasing and the introduction of undersampling artifacts can occur. This can be seen in Fig. 1b-d. Specifically, the Nyquist-Shannon sampling theorem specifies that the grid spacing between k-space samples be

(1) $$\Delta k_{i}=\frac{2\pi }{{\mathrm{FOV}}_{i}},$$

where *i* is one of *x*, *y,* or *z* and FOV_*i*_ denotes the FOV, or image length, in the *i*-direction. The size of the k-space that must be filled with samples separated by Δ*k*_*i*_ is determined by the desired image resolution; the number of image pixels in the *i*-direction must be equal to the number of samples in that *k*_*i*_-direction of k-space. When Cartesian undersampling is applied in one direction with a reduction (or acceleration) factor of *R* (such that only 1/*R* of the total k-space samples are acquired), the effective FOV in the corresponding image dimension is similarly reduced by a factor of *R*. This can be seen by rearranging Eq. (1) to make FOV_*i*_ the subject and selecting the k-space separation in the direction of undersampling, Δ*k*_*i*,under_, to be *R* times larger than the Nyquist distance Δ*k*_*i*,nyq_, giving

(2) $${\mathrm{FOV}}_{i,\mathrm{under}}=\frac{2\pi }{\Delta k_{i,\mathrm{under}}}=\frac{2\pi }{R\Delta k_{i,\mathrm{nyq}}}=\frac{{\mathrm{FOV}}_{i,\mathrm{nyq}}}{R}.$$

As an example, in Fig. 1b undersampling by a factor of *R* = 2 has been applied in the phase encoding direction (*k*_*y*_) such that only every second line of k-space samples is acquired. The resultant image is achieved via an inverse discrete Fourier transform of the undersampled k-space, with unacquired lines zero-filled. Since the FOV in the *y*-direction has been halved, the tessellations of the image are overlapping and multiple copies of the image in different positions are overlaid, a phenomenon known as image aliasing.

Cartesian undersampling can also be performed in two directions, which would induce horizontal as well as vertical aliasing in Fig. 1b. In two-dimensional (2D) imaging, however, this does not provide practical scan acceleration since each phase-encoding line must be traversed by the gradient coils regardless of how many data samples are taken along it. In 3D imaging, however, 2D Cartesian undersampling may be implemented while the acquisitions remain fully sampled in the frequency-encoding (*k*_*x*_) direction. When non-Cartesian sampling trajectories, such as radial (Fig. 1c) or spiral (Fig. 1d) trajectories, are used, none of the sampled points lie at the ideal k-space locations determined by the Nyquist criterion. In this case, the extent of the undersampling is different in different directions, and different along different lines in the same direction. For this reason, no coherent tessellation of the image is shifted into our FOV and overlaid on the center image as was the case for Cartesian undersampling. Instead, incoherent artifacts appear across the resultant images, as can be seen in Fig. 1c and d.

The reconstruction of such images can be achieved via first gridding the data (interpolating the acquired samples to the Cartesian grid), and, for well-chosen gridding functions with a sufficient number of non-Cartesian samples, good quality images may be obtained [4], [5].

To achieve sufficient acceleration to enable high temporal and spatial resolution in dynamic imaging, however, it is impractical to sample the k-space fully. Hence, a great deal of research and innovation has been directed toward reconstruction techniques that are able to produce high-quality dynamic images from undersampled data.

## 3 Early approaches to dynamic image reconstruction

The earliest attempts to implement MRI for dynamic imaging applications aimed to acquire fully sampled 2D k-spaces sequentially [6], [7]. Despite using the fastest acquisition sequences available at the time, they were nevertheless severely limited in both spatial and temporal resolution.

**Fig. 2:**
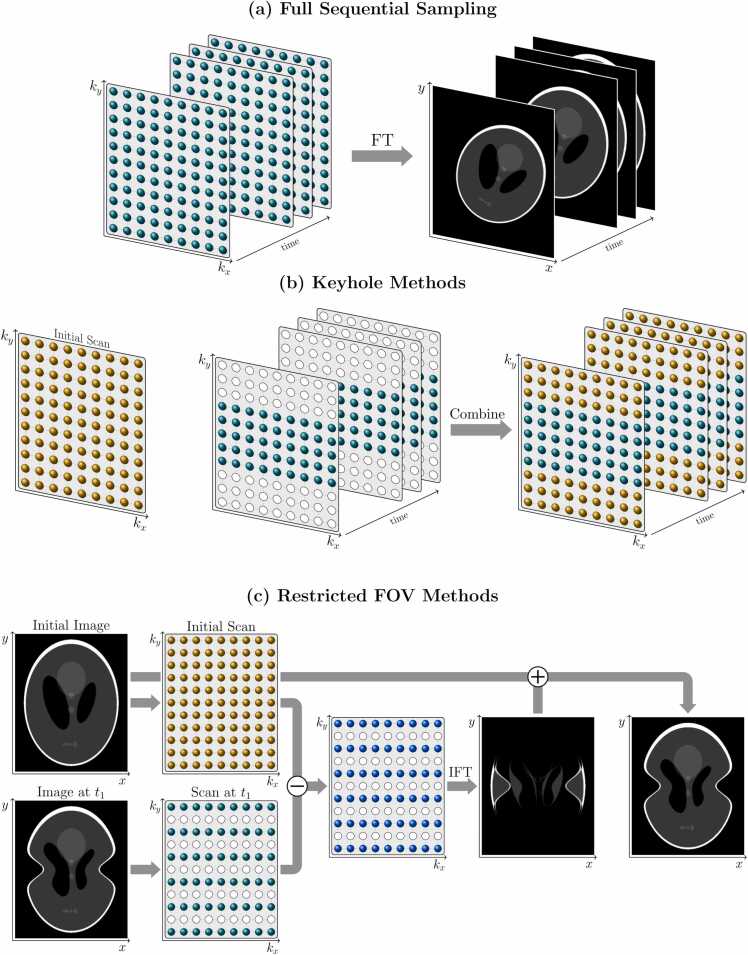
Early reconstruction methods for dynamic imaging. (a) A fully sampled k-space is acquired at each time point. (b) In keyhole methods, the center of k-space is acquired at each time point and combined with the outer-k-space data from an initial scan. (c) The difference between undersampled data at time *t*_1_ and the same samples from an initial scan gives an unaliased difference image since motion is restricted to the center half of the *y*-axis. Combining the initial image with the difference image returns an unaliased image for time *t*_1_. *FT* Fourier transform, *IFT* inverse Fourier transform, *FOV* field of view

Throughout the 1990s, various techniques were proposed to improve the temporal and spatial resolution available in dynamic imaging. In general, these methods made use of the fact that the multiple time-point images acquired during a dynamic scan are very similar; they differ only in changing contrast and/or physiological motion. Therefore, it is reasonable to suspect that the image series can be reconstructed using less data than would be required for a series of completely unrelated images (i.e. less than consecutive, fully sampled k-space acquisitions as illustrated in Fig. 2a).

Keyhole methods [8], [9], for instance, rely on the assumption that most of the dynamic information lies near the center of k-space, thereby corresponding to large-scale and spatially smooth changes. In these methods, the k-space is fully sampled at the beginning of the scan, and only data in a central “keyhole” region of k-space are reacquired at each time point, as shown in Fig. 2b. In reconstructing the time-point images, the data acquired outside the keyhole region from the initial scan are combined with the updated data within the keyhole.

Partial- and half-Fourier approaches [10], [11], [12] similarly use undersampled k-space acquisitions at each time-point and synthesize the remaining unacquired data. In this instance, undersampling is achieved via a truncation of k-space, rather than an increase in the separation of k-space lines, which, as previously discussed, would correspond to a reduced image FOV. For purely real images (without the complex phase inherent in real coil-weighted MR images), k-space is Hermitian, and thus one half of k-space can be determined as the conjugate transpose of the other. In more realistic imaging scenarios, this real-image assumption does not hold exactly, but does provide the basis of several partial-Fourier methods which tend to acquire just over half of the total k-space. For example, in one approach [11], half of the k-space was acquired at each time point alongside a small number of low-frequency lines in the unacquired half of k-space. The remaining higher-frequency lines were then calculated by conjugating and reflecting the acquired lines, under the Hermitian k-space assumption. The complex phase of the underlying image can also be directly accounted for by utilizing an initial fully sampled scan to calculate magnitude and phase calibration constants which relate the Hermitian-symmetric pairs in the two halves of k-space [12]. By assuming these remain constant throughout the scan, only half the k-space needs to be acquired at each time point, with the calibration constants used to synthesize the other half.

Another class of methods assumes that the motion is restricted to a particular region of the image [13], [14], such as the heart in cardiac cine. If the dynamic region occupies only one *R*th of the total image FOV, only one *R*th of the k-space lines need to be reacquired at each time point to reconstruct the dynamic region. Without further correction, however, time-point images reconstructed from such data would be affected by aliasing of the static image region, since the k-space is now undersampled relative to the overall FOV. Different approaches to alleviating this difficulty were proposed. For example, in one subtraction-based technique [13], the acquired k-space lines at each time point were subtracted from the same lines in a fully sampled initial scan. The inverse Fourier transform of this difference data was taken to provide a reduced-FOV difference image which was correctly positioned within the full FOV using prior knowledge of the dynamic region location. Finally, the difference image was added to the fully sampled static image to yield the full-FOV dynamic image for that time point. This approach is depicted in Fig. 2c. An alternative proposal was to instead construct a k-space data set corresponding only to the static region of the image [14]. This was achieved via the temporal averaging of all k-space lines with pixel values in the dynamic image region set to zero. Subtraction was again used to determine the dynamic and reduced FOV in the dynamic region, with the dynamic and static images combined to yield the full-FOV image at each time point. By averaging over all data to achieve their static image, however, an increase in the signal-to-noise ratio (SNR) was realized.

## 4 Parallel imaging

A critical development in the history of MRI scan acceleration was the introduction of parallel imaging techniques. In parallel imaging, k-space data are acquired simultaneously in every coil of a multi-coil array [15], as opposed to in a single volume coil, as had been common previously. Since the k-space location of the RF signal received in a coil is determined by the gradient pulse sequence, the same k-space location is acquired in all coils simultaneously, and no increase in scan time is required to acquire *N*_*c*_ copies of the k-space, each corresponding to one of the *N*_*c*_ coils in the array. Together with providing improvements in SNR, an advantage of this approach is that rather than producing identical (and thereby redundant) copies of k-space, each set of k-space data instead corresponds to the image multiplied by the unique sensitivity profile of the coil that it was acquired with. That is, it consists of discrete samples of the Fourier transform of $S_{j}\left(x\right)\rho \left(x\right)$, instead of just the image $\rho \left(x\right)$, where $S_{j}\left(x\right)$ is the sensitivity profile of the *j*th coil. Essentially, each coil only “sees” a limited part of the imaging region close to its position. In this way, parallel imaging arrays provide additional spatial encoding of the k-space data, which can be used to partially replace the Fourier spatial encoding and hence accelerate scan times.

Many algorithms have been proposed to take advantage of the additional information provided by multi-coil arrays [16], [17], [18], [19], [20], [21], [22], [23], [24], [25], [26]. Of these, we will consider three of the most popular approaches, SENSE [16], GRAPPA [24], and iterative self-consistent parallel imaging reconstruction (SPIRiT) [25], in detail. These methods have found widespread use in MR image reconstruction. Additionally, they form the basis of many methods specifically designed for dynamic applications, as is discussed later. However, to introduce parallel image reconstruction, we begin instead with the parallel imaging with localized sensitivities (PILS) [17] technique. PILS has strict coil sensitivity requirements which mean it is often not applicable in real-world situations, but it nevertheless provides an intuitive explanation of how multi-coil arrays can provide scan acceleration while avoiding the introduction of aliasing artifacts.

### 4.1 PILS

In PILS [17], a 2D acquisition is accelerated in the phase-encoding (*k*_*y*_) direction by a reduction factor of *R*, such that only every *R*th k-space line is acquired and the scan time is similarly reduced by a factor of *R*. To achieve this acceleration and still obtain an unaliased full-FOV image, the multi-coil array must contain at least *R* coils (*N*_*c*_ ≥ *R*), and each coil sensitivity profile must be localized to a region with a height no greater than FOV_*y*_/*R*, where FOV_*y*_ is the full image FOV in the *y*-direction. Beyond this region, the coil sensitivity must be zero. Additionally, the position of the center of each sensitive region must be known.

**Fig. 3:**
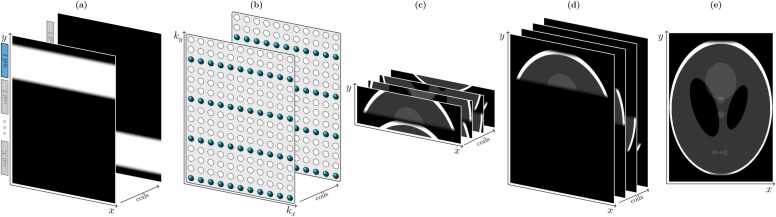
Stages in a PILS acquisition and reconstruction. (a) Localized coil sensitivity maps consistent with the requirements for a PILS reconstruction. (b) The undersampled k-space acquired with each coil. (c) The inverse Fourier transform of each k-space gives a series of unaliased images with a reduced FOV in the *y*-direction. (d) The reduced-FOV images shifted to the correct position in the full FOV. (e) The resultant unaliased image formed via a sum-of-squares combination of the coil images in (d). *PILS* parallel imaging with localized sensitivities, *FOV* field-of-view

An example of coil sensitivity profiles consistent with this requirement for a four-coil array can be seen in Fig. 3a, and the corresponding k-space sampling pattern for *R* = 4 is illustrated in Fig. 3b. When the inverse Fourier transform is applied to each k-space, without zero-padding, the vertical FOV is also reduced by factor of *R* in accordance with Eq. (2). As discussed previously, this would normally lead to image aliasing. Now, however, as each coil image is zero outside of the localized region, the height of the non-zero region of the image is equal to or less than the reduced height of the image (FOV_*y*_/*R*). Therefore, the image wraparound effect induced by k-space undersampling, while still present, does not cause multiple parts of the image to be overlaid at the same position, as can be seen in Fig. 3c. The known *y*-value can then be used to position each reduced-FOV coil image correctly within the full FOV (Fig. 3d). Finally, the coil images can be combined (using, e.g., a pixel-wise sum-of-squares approach) to yield a full-FOV image covering the entire imaging region of interest free from any aliasing effects (Fig. 3e).

### 4.2 SENSE

A more flexible framework is provided by the SENSE technique [16], which acts in image space to “unfold” an aliased image. SENSE is compatible with arbitrary coil array configurations, provided that the corresponding coil sensitivity profiles introduce sufficient spatial encoding to the k-space data. This only occurs when there is sufficient variation between the profiles; if the coil sensitivity values are too similar, a SENSE reconstruction may fail.

A successful SENSE reconstruction also requires that coil sensitivity profiles be accurately known. In practice, coil sensitivity maps are dependent on the object being imaged and need to be redetermined for every scan. A fast low-resolution calibration scan is often performed prior to the undersampled SENSE, enabling low-resolution images to be reconstructed for each coil in the array. Dividing each individual coil image by an un-weighted image (formed, for instance, using a full body coil, or via sum-of-squares [16], [27], or adaptive combination [28] of the individual coil images) effectively removes the underlying object from the images and provides acceptable coil profiles. Potential noise in profiles thus obtained may be reduced through local polynomial fitting [16], [27]. Since the profiles tend to be smoothly varying, the low resolution of the calibration scan is sufficient to capture their spatial variation. However, movement of either the patient or the coils between the calibration and the scan can introduce a mismatch between the estimated coil sensitivities and those corresponding to the undersampled data [29], and lead to artifacts in the reconstructed image. Auto-calibrated techniques [29], [30] such as modified SENSE [31] have been proposed to mitigate this issue. These techniques utilize additional k-space lines acquired with full sampling at the center of k-space to estimate the sensitivity profiles. Since the calibration data are acquired simultaneously to the undersampled SENSE data, motion occurring between the acquisitions is eliminated.

**Fig. 4:**
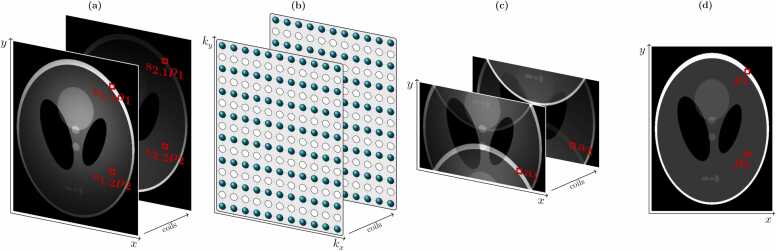
Stages in a SENSE reconstruction. (a) Coil-weighted images, as “seen” by the coils in the array. (b) An undersampled k-space is acquired with each coil. (c) Taking the inverse Fourier transform of each k-space gives a series of aliased images. (d) SENSE reconstruction “unfolds” the aliased image to give the full-FOV image. *SENSE* sensitivity encoding, *FOV* field-of-view

As depicted in Fig. 4, in SENSE, the inverse Fourier transform of each undersampled coil-weighted k-space is taken to yield a set of *N*_*c*_ images, all of which are affected by aliasing. In the example shown, undersampling with a reduction factor of *R* = 2 has been implemented in the phase encoding (*k*_*y*_) direction only. In general, however, SENSE is applicable to Cartesian undersampling in multiple directions and higher reduction factors, provided the overall reduction factor is less than or equal to *N*_*c*_, the total number of coils in the phase encoding direction. Each pixel in each of the aliased images is formed via the superposition of multiple sensitivity-weighted pixels from the full-FOV coil-weighted images. This is highlighted in Fig. 4; the red boxes in Fig. 4a indicate the positions of two pixels in the full-FOV images, *ρ*_1_ and *ρ*_2_, which alias to the same position in the reduced-FOV images, labeled *a* and indicated by the red boxes in Fig. 4c.

The relationships between unaliased pixel values and aliased coil-weighted image pixels can be expressed in sets of linear equations, as detailed in Appendix A.2. Sets of linear equations such as these can be solved provided the number of equations is greater than or equal to the number of unknowns. Here, there is one equation for each coil in the array, and each set of equations contains all the pixels *R* that are aliased to the same position in the reduced-FOV image (the unknowns), hence the requirement for the number of coils to be equal or greater than the acceleration factor.

When each of the sets of equations is solved, the calculated pixel values *ρ*_*n*_ can be positioned appropriately to recover a full-FOV image without aliasing, as illustrated in Fig. 4d.

Generally, however, and particularly for non-uniform sampling patterns, a large system of equations will be obtained, and thus a more computationally efficient iterative scheme is often used to reconstruct images in SENSE [19]. The problem becomes to iteratively solve

(3) b=FSρ

for ***ρ***, where ***ρ*** is the vector of image pixels, **b** is a vector of the data acquired in all coils, *S* is a matrix of coil sensitivities, and *F* is a Fourier transform operator. More generally, *F* and *S* can be replaced by an encoding operator *E* which contains any operations required to transform the solution image to the acquired data. Within this framework, non-Cartesian trajectories, such as radial and spiral, can easily be incorporated since the implementation of *E* can utilize either a non-uniform FFT algorithm or k-space gridding [4] between the Cartesian and acquired grids at each iteration.

Noise amplification, and a corresponding reduction in SNR, is an inevitable result of SENSE and other parallel-imaging-based acceleration methods. The SNR of an undersampled SENSE reconstruction is related to the SNR of a fully sampled acquisition via the equation

(4) $${SNR}_{\mathrm{under}}(x,y)=\frac{{SNR}_{\mathrm{fully}}\left(x,y\right)}{g\left(x,y\right)\sqrt{R}}.$$

Here *g* is the *g*-factor (geometry factor), which is often used to characterize the spatial distribution of the noise amplification and depends on the geometry of the coil array [16], [32]. Eq. (4) reveals that the loss in SNR is also dependent on the square root of the acceleration factor *R*, since with higher acceleration fewer samples are available from which to reconstruct the image.

The SENSE equations can become ill-conditioned when there are areas of poor k-space coverage (such as the corner of a rectangular k-space FOV when a radial or spiral trajectory is used) or if there is insufficient SENSE due to insufficiently distinct coil sensitivity profiles [19], [33]. Mathematically, ill-conditioned problems are characterized by the property that small changes to their input (the k-space data, in the present case) may result in large changes to their output (the reconstructed images). In such scenarios, SENSE can become sensitive to, and amplify, noise in the acquired data. Various regularization techniques can be employed to restrict this noise amplification and improve image quality. For instance, k-space can be filtered to reduce the contribution of values found in regions with poor coverage [19] or a singular value decomposition (SVD)-truncation can be applied to the encoding operator *E* [34]. Alternatively, the iterative formulation allows regularization functions to be added to $∥E\rho -b{∥}_{2}^{2}$, the SENSE objective, and minimized during the iteration process. A simple Tikhonov objective function, calculated as the norm of the image pixel values, has been demonstrated to reduce image noise [33], [35], while more complicated penalty functions are often utilized within a similar framework in CS techniques, as discussed further in Section 6.

While primarily introduced here as background for the more-advanced dynamic reconstruction methods discussed in later sections, SENSE itself has been applied on a frame-by-frame basis to accelerate real-time cardiac imaging [36], [37]. Such applications do not take advantage of the temporal redundancies intrinsic to dynamic MRI datasets, and as such a trade-off between spatial and temporal resolution remains a limiting factor.

### 4.3 GRAPPA

Distinct from SENSE and other methods that operate directly in image space to “unfold” aliased images, are the class of parallel imaging algorithms that attempt to recover unacquired data in k-space [24], [25], [26]. Among these, GRAPPA [24] is the most common. Unlike SENSE, GRAPPA is auto-calibrating, meaning that accurate coil sensitivity profiles are not required as an input to the method. This may be particularly advantageous in imaging scenarios where movement occurs between the sensitivity calibration scan and the undersampled scan, although, as mentioned auto-calibrated extensions of SENSE have also been developed. In GRAPPA, the requirement for sensitivity profiles is replaced by the need for an auto-calibration signal (ACS), which generally consists of a fully sampled set of k-space lines around *k*_*y*_ = 0 (assuming undersampling in the *k*_*y*_ direction). The idea underlying GRAPPA is that multi-coil k-space data are correlated, since each coil’s k-space corresponds to the same physiological image modulated by a generally smooth coil sensitivity profile. Specifically, GRAPPA proposes that each unacquired k-space sample may be reliably synthesized by a linear combination of the acquired k-space samples in its multi-coil k-space neighborhood, and, furthermore, that the weights used in this linear combination are constant throughout k-space.

The first assumption builds on ideas introduced in the simultaneous acquisition of spatial harmonics (SMASH) [21] method, and its subsequent auto-calibrated extensions[22], [23]. SMASH utilizes linear combinations of a single k-space line acquired in different coils to synthesize unacquired neighboring lines. The linear combination weights are chosen such that the combined coil sensitivity profile closely matches a spatial harmonic which modulates the exponential term in the integrand of the Fourier transform (Eq. (A.1)) so that it now corresponds to an adjacent k-space position, as described in Appendix A.3.

In GRAPPA, this idea is extended and made more robust by including k-space samples from multiple locations surrounding the target location, and by synthesizing the missing samples in every coil rather than just in a k-space corresponding to the final image unmodulated by coil sensitivities.

**Fig. 5:**
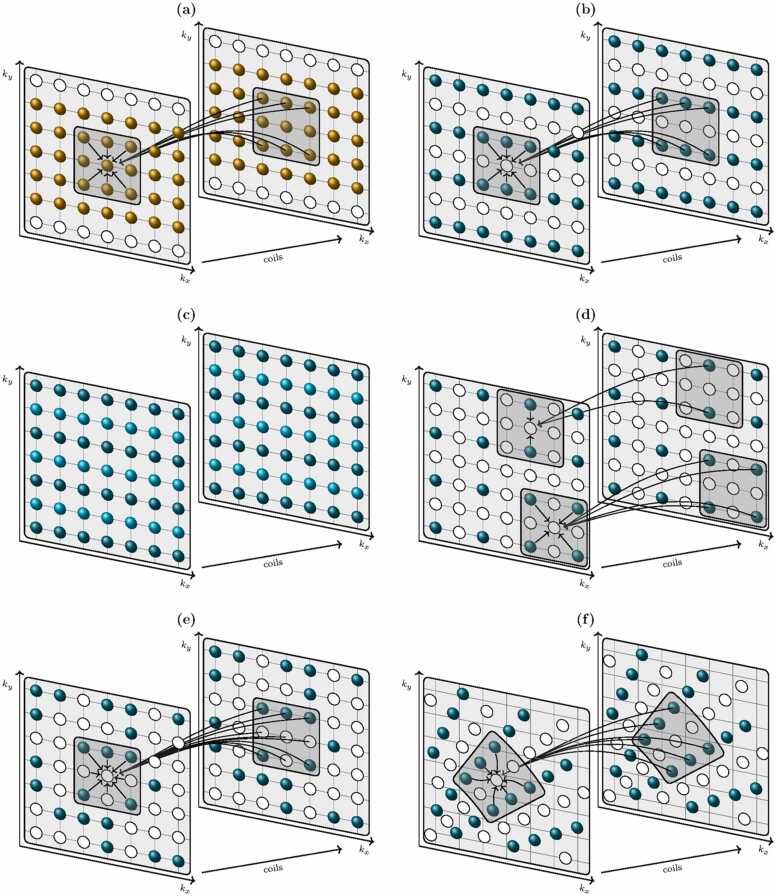
Kernel calibration and k-space synthesis for GRAPPA and SPIRiT reconstructions. (a) Kernel calibration in the ACS region at the center of k-space. (b) k-Space synthesis with GRAPPA for a two-fold undersampling in the *k*_*y*_ direction. (c) Fully sampled multi-coil k-space with unacquired samples synthesized using GRAPPA. (d) GRAPPA applied to 2D undersampling where different neighborhood acquisition patterns exist for different positions in k-space. (e) A SPIRiT kernel for an arbitrary sampling pattern with both acquired and unacquired samples contributing to the synthesis of an unacquired sample. (f) GRAPPA applied to a radial sampling trajectory where the neighborhood takes the shape of a truncated wedge. *GRAPPA* generalized autocalibrating partially parallel acquisitions, *SPIRiT* iterative self-consistent parallel imaging reconstruction, *ACS* auto-calibration signal, *5D* five-dimensional

Fig. 5a and b depicts the k-space geometry of a standard GRAPPA reconstruction with two-fold undersampling in the *k*_*y*_ direction. Near the center of k-space, shown in Fig. 5a, the k-space is fully sampled due to the acquisition of ACS lines, whereas in other regions, depicted in Fig. 5b, only every second phase-encoding line is acquired. In these figures, the shaded region represents the multi-coil k-space neighborhood of a k-space sample to be synthesized. As indicated by the arrows in Fig. 5b, all the acquired samples that sit within this neighborhood contribute to the synthesis of the unacquired sample via a linear combination which utilizes a calibrated kernel of weights. A mathematical description of the GRAPPA synthesis is included in Appendix A.4.

To implement GRAPPA and synthesize the missing samples in every coil, the kernel of weights, *g*, must first be calibrated using the ACS lines. As indicated by the arrows in Fig. 5a, the calibration process utilizes the same multi-coil neighborhood geometry as the synthesis step, but here both the target point being synthesized and the source points contributing to the synthesis are known, since the ACS region is fully sampled. By equating the known value of each target point with the value that would be produced were it to be synthesized from the neighboring source points with GRAPPA, an equation is constructed for every possible kernel position within the ACS region. The resultant system of equations is solved to find the weights, often in a least-squares manner since the number of equations (determined by the number of viable kernel positions) ideally exceeds the number of weights, increasing robustness to noisy data.

Once the kernel of weights has been calculated, the unacquired samples are synthesized across every coil, creating a fully sampled multi-coil k-space, as illustrated in Fig. 5c. From this, coil images can be calculated using the standard inverse Fourier transform operation and combined using, for example, a sum-of-squares technique.

In certain cases, it can be advantageous to undersample in more than one direction. For example, for a 3D acquisition, undersampling can be implemented in both the phase-encoding (*k*_*y*_) and slice (*k*_*z*_) directions. GRAPPA is readily applicable to multi-dimensional undersampling.[25] However, as can be seen in Fig. 5d, which depicts the GRAPPA kernels for a 2D undersampling scheme with two-fold undersampling in each of the *k*_*x*_- and *k*_*y*_-directions, different neighborhood acquisition patterns arise for different samples being synthesized. In these cases, a separate set of weights must be calibrated and applied for each pattern.

Variants of GRAPPA have also been developed for non-Cartesian trajectories [38], [39], [40], [41], [42], [43], [44], including, for instance, radial [38], [39], [40], [41], spiral [42], [43], and Periodically Rotated Overlapping ParallEL Lines with Enhanced Reconstruction (PROPELLER) [44] acquisitions. These applications pose the additional complication that the length and direction of the displacement between neighboring samples vary with position. For example, in a radial trajectory, the rectangular kernel geometry from Cartesian GRAPPA is replaced with a truncated wedge, as is illustrated in Fig. 5f. The displacement between the target point at the center of this kernel and a neighboring source point will increase as the radius of the kernel center increases, while the displacement vector will be rotated as the angular position of the kernel changes. In such non-Cartesian circumstances, Eqs. (A.10)–(A.12) do not apply and the kernel of weights cannot be applied throughout k-space. Initial implementations of radial GRAPPA [38], [39] required a fully sampled calibration dataset and then utilized segmentations in both the radial and azimuthal directions. Within each segment, the differences were assumed to be small, allowing a standard calibration where the radial data are treated as Cartesian, and separate weights were calibrated and used in each segment. More accuracy can be obtained by defining a separate segment for each kernel position, with that kernel at its center [40]. A trade-off exists between the benefits of smaller calibration segments (which more closely satisfy the invariant displacement vector assumption and reduce blurring) and the increased number of viable kernel positions allowed by larger segments, which tend to reduce noise. Additional kernel instances can be achieved for smaller calibration segments in dynamic imaging applications by obtaining multiple fully sampled radial k-spaces before the undersampled scan [40], [43]. Similarly, calibration data from multiple slices can be used together in a 3D stack-of-stars acquisition [41].

Self-calibration schemes for non-Cartesian GRAPPA have also been proposed [45], [46], [47], [48], [49]. For example, the radial self-calibrated approach [45] calculates weights using larger kernels on the undersampled radial acquisitions (with correspondingly increased displacements between target and source points). The required weights for smaller kernels positioned on the full radial grid are then interpolated from the weights calculated at various radii but the same angular position. A conceptually similar approach involves searching the acquired radial k-space for similar sampling patterns to each needed for the synthesis of an unacquired sample [46]. Self-calibration for arbitrary non-Cartesian trajectories has been realized through the use of a Cartesian calibration region (gridded from a highly sampled region of the k-space) [47], [48]. This can be then used to grid or interpolate multiple instances of each neighborhood sampling pattern that appear in the undersampled trajectory for calibration.

As was the case for SENSE, GRAPPA can be applied frame-by-frame to dynamic cardiac data [50] without exploiting through-time correlations. However, greater efficiency can be realized from GRAPPA-based reconstructions of dynamic data by constructing calibration regions from neighboring temporal frames (as mentioned above for radial GRAPPA) or by applying GRAPPA-like concepts to a high-dimensional spatio-temporal domain. These approaches are discussed further in Section 5.

### 4.4 SPIRiT

The SPIRiT technique further generalizes GRAPPA by avoiding the requirement of different calibration kernels for every acquisition pattern, and thereby permits the use of arbitrary Cartesian k-space undersampling. This is achieved by constructing a single kernel with weights corresponding to every sample in the multi-coil neighborhood, regardless of whether or not those samples have been acquired. Fig. 5e illustrates the concept; with an arbitrary Cartesian undersampling pattern the target point at the center of the kernel in the first coil has both acquired and unacquired samples in its multi-coil neighborhood, and the target point is synthesized (as indicated by the arrows) with a linear combination of all these samples. However, since the unacquired samples are unknown, and must also be synthesized using the same procedure, the synthesis step cannot be immediately implemented following kernel calibration as it was with GRAPPA. Instead, an iterative solution is found to satisfy

(5) $$\arg\underset{b}{\min}∥Db-b_{\mathrm{acq}}{∥}_{2}^{2}+\lambda ∥\left(G-I\right)b{∥}_{2}^{2},$$

where **b** is the vector of synthesized fully sampled multi-coil k-space data to solve for, **b**_acq_ is a vector of the acquired data, *D* is an undersampling operator which selects only the acquired points from the fully sampled k-space, *λ* is a penalty weighting, *I* is the identity, and *G* is a matrix constructed from the kernel elements which applies the kernel convolution to each coil’s k-space. Eq. (5) consists of two constraints; the first term requires that the synthesized data matches the acquired data at those locations where data are acquired, while the second term requires the solution to be consistent with the kernel, such that applying the linear-combination synthesis to the solution (where unlike Fig. 5e all neighboring samples now have values) returns the same multi-coil fully sampled k-space.

The SPIRiT formalism can also be adapted for non-Cartesian acquisitions by simply replacing the undersampling operator *D* with an operator that interpolates the fully sampled data to the off-grid locations of the acquired non-Cartesian samples.

## 5 Exploiting temporal redundancy

During the period when pioneering techniques in parallel MRI were being introduced, so too were a series of reconstruction methods designed specifically for dynamic imaging applications. Just as correlations in multi-coil k-space data were exploited to accelerate scans with parallel MRI, these methods exploit the correlations present in the temporal dimension of dynamic scans.

As such, these methods often consider a higher dimensional x-t domain, where t represents the temporal dimension of the scan and x represents the spatial dimensions (generally two or three). The goal of image reconstruction becomes to fully reconstruct the image in x-t space such that a full FOV image without artifacts exists for every discrete value of t, as determined by the desired frame-rate of the scan. From this concept follows other useful parameter domains such as k-t space (the k-space at each time point), and x-f and k-f space, where f is the temporal frequency. The latter two can be obtained from x-t and k-t space, respectively, via a one-dimensional (1D) Fourier transform applied in the temporal dimension.

Previous works had explored the theory underlying spatio-temporal sampling [51], [52], [53], [54], but the first prominent application of these ideas to MRI was the unaliasing by Fourier-encoding the overlaps using the temporal dimension (UNFOLD) [55] method. UNFOLD allows undersampling by separating aliased components in the temporal frequency dimension and can also be combined with parallel imaging [56], [57], [58]. Building on UNFOLD, k-t broad-use linear acquisition speed-up technique (BLAST) and k-t SENSE [59] exploit high dimensional aliasing in x-f space to achieve higher acceleration or reduce remaining artifacts from parallel imaging. In this section, we discuss the UNFOLD and k-t SENSE approaches, and also consider the temporal GRAPPA (TGRAPPA) [60] and k-t GRAPPA [61] methods which extend GRAPPA for dynamic applications.

### 5.1 UNFOLD

Like SENSE, the UNFOLD [55] method acts in image space to unfold aliased images. The manner in which it does so is also similar to SENSE; both UNFOLD and SENSE are able to separate individual pixel contributions to an aliased pixel since multiple aliased images are formed via linear combinations of the unaliased pixels with different combination weights. In SENSE, each aliased image corresponds to a single coil in the array, and the aliased pixel values are modulated by the coil sensitivities. In UNFOLD, each aliased image instead corresponds to a time point in a dynamic acquisition, and the aliased images correspond to different undersampling trajectories which are used at each time point.

**Fig. 6:**
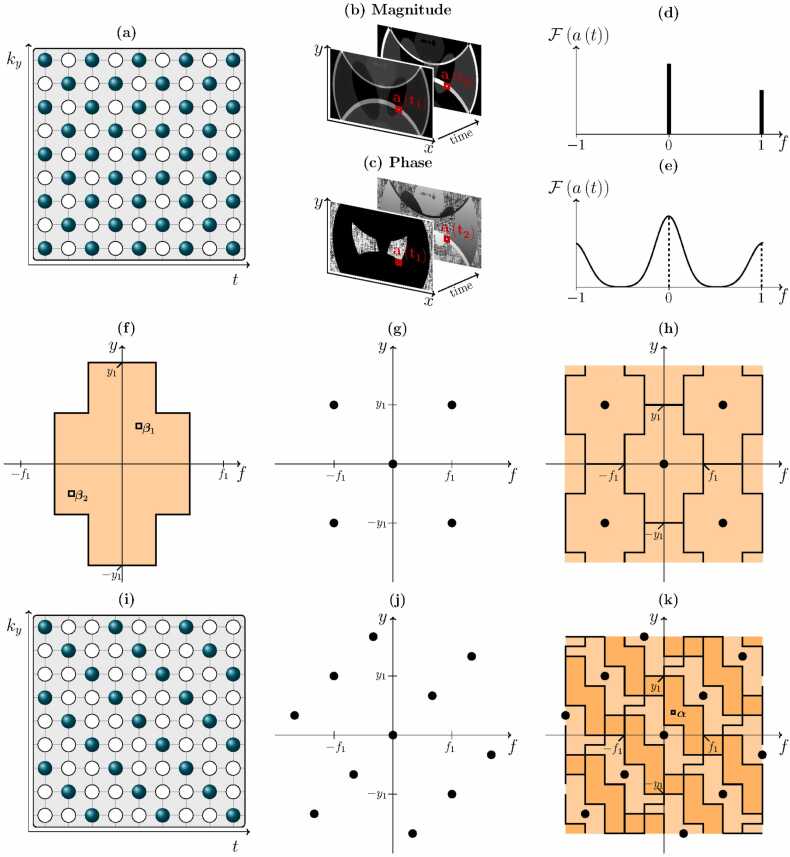
Diagrams depicting k-t acquisition patterns, x-f support regions, and x-f aliasing for methods exploiting temporal redundancies. (a) A staggered two-fold-undersampled k-t acquisition pattern for UNFOLD. Each line is fully sampled in the *k*_*x*_-direction (not pictured). (b) and (c) Magnitude and phase of aliased images produced at odd and even time points from the trajectory in (a). (d) and (e) The temporal frequency spectrum for an arbitrary pixel acquired with the trajectory in (a) when motion is (e) and is not (d) present. (f) An x-f support region in UNFOLD, assuming the center half of the *y*-axis requires twice the temporal frequency information to describe the motion as than outer half does. (g) The PSF in x-f space associated with the k-t acquisition pattern in (a). (h) Tessellation of the x-f support region shown in (f) due to the k-t acquisition pattern in (a). (i) A three-fold-undersampled k-t acquisition pattern for k-t SENSE. (j) The PSF in x-f space associated with the k-t acquisition pattern in (i). (k) Aliasing of the support region shown in (f) due to the k-t acquisition pattern in (i). *UNFOLD* unaliasing by Fourier-encoding the overlaps using the temporal dimension

As an example, consider the two-fold undersampled 2D Cartesian acquisition pattern utilized in the original presentation of UNFOLD [55]. At each time point every second phase-encoding line is acquired, but the acquisition alternates between odd and even lines. This staggered sampling pattern is depicted in Fig. 6a, where the frequency encoding (*k*_*x*_) direction (not shown for simplicity) is assumed to be fully sampled. As we have seen previously, two-fold undersampling causes image aliasing such that two pixels are superimposed at every location in the reduced FOV image at every time point. However, in accordance with the Fourier shift theorem, the aliased image from the even k-space lines will not be identical to that from the odd k-space lines. Since the sampling function $A\left(k_{x},k_{y}\right)$ has been shifted in the *k*_*y*_-direction for every second time point, a phase shift is applied to the corresponding PSF. A given pixel in the aliased images thus contains contributions from the same pixels from the full FOV image, but these have been modulated by different phases, as can be seen in Fig. 6b and c where the magnitude and phase of the images at each time point are shown separately.

If the image is static, as is the case in Fig. 6b and c, then each aliased pixel value alternates between the sum and the difference of its two constituent pixels (as the phase modulation experienced by the neighboring image tessellation alternates between *e*^*iπ*0^ = 1 and *e*^*iπ*^ = −1):

(6) $$a\left(t_{\mathrm{even}}\right)=\rho_{1}+\rho_{2}\;a\left(t_{\mathrm{odd}}\right)=\rho_{1}-\rho_{2}.$$

Therefore, taking the Fourier transform in the temporal dimension for a given aliased pixel yields exactly two non-zero components: the direct current (DC) component (attributable to *ρ*_1_) and the Nyquist frequency (attributable to *ρ*_2_). This is shown in Fig. 6d and permits a more flexible view of UNFOLD which can accommodate motion; rather than simply unfolding aliased images, UNFOLD separates aliased information in the temporal frequency direction.

When, as is the case in a dynamic imaging scenario, the constituent pixels contain motion, the discrete frequency spikes will be replaced with broader spectra, as shown in Fig. 6e. The bandwidth of these curves is determined by the motion present in the pixel itself; the more rapid and aperiodic the motion, the larger the range of temporal frequencies required to represent it accurately. When the temporal bandwidths are sufficiently narrow such that they do not overlap, as is the case in Fig. 6e, UNFOLD can be used to separate the aliased pixels by filtering in x-f space. Within this framework, it can be seen that UNFOLD can be applied to arbitrary undersampling schemes, provided the sampling pattern is altered each frame in a periodic manner, there is sufficient bandwidth to permit the same number of non-overlapping pixel spectra as there are frames in one period and that the accumulated sampling pattern over a full period is sufficient to provide an image free from aliasing and artifacts.

Additionally, regions of the image where more motion is expected, such as the heart in cardiac imaging, are likely to require wider bandwidths, but these can be stacked in x-f space alongside more-static regions with narrower bandwidths. A simple framework for visualizing the potential acceleration is provided by examining the support regions in x-f space [62]. Fig. 6f depicts a cross-shaped x-f support region, consistent with an imaging scenario where a dynamic region (requiring a larger range of temporal frequencies) is present in the center half of the *y*-axis. Now, since x-f space is formed via the Fourier transformation of the k-t domain in every dimension, the PSF of the staggered acquisition pattern in Fig. 6a exists in x-f space and is depicted in Fig. 6g. As shown in Fig. 6h, the replications of the cross-shaped support region induced by this PSF tessellate without overlap.

UNFOLD can therefore be implemented as a filtering operation in the x-f domain. Exactly one copy of the support region is retained, and everywhere else is set to zero.

UNFOLD may also be combined with parallel imaging algorithms such as SENSE [56], [57], [58] and GRAPPA [57], where it can be used to either increase the acceleration factor, remove residual artifacts remaining after reconstruction, or a combination of the two. Temporal filtering SENSE (TSENSE) [56] and UNFOLD-SENSE [58] are two methods that combine UNFOLD with SENSE. While differing in the details of their implementation, a feature of both is that through UNFOLD-like temporal filtering they allow the estimation of time-varying sensitivity maps which can be utilized in the SENSE reconstruction. Such adaptive coil maps preclude the need for an initial reference scan and can accommodate relatively slow variations (e.g. respiratory motion).

### 5.2 k-t SENSE and k-t BLAST

The k-t acquisition scheme used in UNFOLD avoids aliasing by ensuring support region replicates do not overlap in the x-f domain, and then applies a filter in that domain to extract just one copy. Effectively, SENSE-like unaliasing concepts are used to unalias x-t images by separating them in the temporal frequency dimension f. Building on these ideas, the k-t BLAST (single coil) and k-t SENSE (multi-coil array) methods go further; by permitting higher acceleration factors that do lead to aliasing in the x-f domain, k-t SENSE reconstructs the unaliased time series of images by applying a SENSE-like unfolding algorithm in the higher dimensional space.

In k-t SENSE, the staggered acquisition pattern of UNFOLD is utilized with higher acceleration factors. This is depicted in Fig. 6i where an acceleration of *R* = 3 each frame is applied, leading to closer peaks in the PSF (Fig. 6j). In the x-f domain, this leads to the overlapping of the same support region which produced no x-f aliasing for two-fold undersampling (Fig. 6k). The arbitrary point *α*, indicated in the aliased x-f domain, is seen to be formed from the overlap of the points labeled *β*_1_ and *β*_2_ in Fig. 6f.

In general, an arbitrary number of points may alias to the same position in x-f space, and a set of linear equations describing these points can be constructed (see Appendix A.5). These share the same structure as the SENSE equations and can be solved in a similar way. For k-t BLAST, and k-t SENSE when the number of coils is less than the number of x-f points aliasing to the same position, the equations are underdetermined and no unique solution exists. For this reason, an initial low-spatial-resolution training scan is used to estimate a matrix *M*^2^ containing the expected magnitudes of each *β*. Alternatively, the training scan can be avoided by utilizing an auxiliary TSENSE [56] reconstruction to generate the training data [63].

A solution is found that ensures each *β* matches its expected signal magnitude, with the vector of values ***β*** found by solving

(7) $$\beta =\underset{\_}{\beta }+{\left(S*\Psi^{-1}S+M^{-2}\right)}^{-1}S*\Psi^{-1}\left(\beta_{\mathrm{alias}}-S\underset{\_}{\beta }\right),$$

where *S* is a matrix of coil sensitivities, Ψ is a matrix containing the covariance of noise levels across the coils, $\underset{\_}{\beta }$ is a baseline estimate of ***β***, and * denotes the conjugate transpose. When k-t SENSE was first proposed, the baseline estimate was obtained using temporal averaging. However, this results in a number of nulled temporal frequencies, since the DC component (equivalent to the temporal average) is aliased to *R*-1 higher frequencies, and thus these frequencies are also removed when the baseline subtraction is performed [64]. Alternative baselines formed using TGRAPPA [64] (discussed below) or reduced-encoding imaging by generalized-series reconstruction (RIGR) [65], [66] techniques have been proposed; these approaches are able to recover the nulled frequency information and result in reduced artifact power. When arbitrary non-Cartesian sampling schemes are used or the time-invariant coil sensitivity assumption cannot be met, the equation becomes

(8) $$\beta =\underset{\_}{\beta }+{\left(E*\Psi_{k,t}^{-1}E+M^{-2}\right)}^{-1}E*\Psi_{k,t}^{-1}\left(b_{k,t,\gamma }-E\underset{\_}{\beta }\right),$$

where *E* is a forwards operator similar to that which models the acquisition in iterative SENSE (Eq. (3)). Here, *E* explicitly incorporates the sampling pattern and applies coil-sensitivity weights to every time-point, Ψ_*k*,*t*_ is the noise covariance matrix in k-t space and **b**_*k*,*t*,*γ*_ is the vector of acquired k-t samples in the *γ*th coil.

### 5.3 TGRAPPA and k-t GRAPPA

TGRAPPA and k-t GRAPPA have both been proposed as extensions to GRAPPA for dynamic imaging applications. In TGRAPPA [60], a staggered sampling pattern, as used in UNFOLD and k-t SENSE, is implemented to avoid the requirement of acquiring ACS lines; the lines near the center of k-space across neighboring temporal frames are collected and used together as the GRAPPA calibration region. Once the kernel is calibrated, however, the GRAPPA k-space synthesis proceeds normally and separately for each time point.

The k-t GRAPPA method [61] explicitly incorporates the higher dimensional k-t domain by applying the GRAPPA kernel in the k-t domain. An unacquired line is synthesized via a linear combination of acquired lines which include both spatial neighbors, acquired at the same time point as the line being synthesized, and temporal neighbors, acquired at the same k-space location before or after the time the line being synthesized corresponds to. To calibrate the k-t GRAPPA kernel, an ACS region in k-t space, similar to that seen for regular GRAPPA in Fig. 5a, is obtained by fully sampling the center of k-space at each time point.

## 6 Compressed sensing and low-rank methods

CS was first proposed for MRI reconstruction in the mid-2000s [67], [68], [69] and has become a popular technique for many applications. The success of CS lies in the fact that medical images do not consist of random independent pixels stacked side-by-side. Rather, they exhibit clear spatial correlations and, in particular, have sparse representations upon transformation to certain domains (meaning they can be represented by a number of elements in these domains that is far fewer than the number of pixels or voxels in the image). This fact can be used as prior information to regularize the ill-conditioned inverse problem that is undersampled MR reconstruction, provided the acquisition scheme would generate incoherent aliasing artifacts (as seen for radial and spiral sampling in Fig. 1c and d, but not for uniform Cartesian undersampling as seen in Fig. 1b). The general CS MRI reconstruction problem can be expressed as

(9) $$\arg\underset{\rho }{\min}∥E\rho -b{∥}_{2}^{2}+\lambda ∥\Psi \rho {∥}_{1},$$

where Ψ is the sparsifying transform, *λ* is a penalty weighting, and the first term, which involves the encoding operator *E*, the acquired data **b** and the image ***ρ***, forces the sparse solution to be consistent with the acquired data. The *L*_1_-norm is applied to the sparse domain since its minimization promotes sparsity. If desired, multiple sparsity terms can be included in Eq. (9) with different penalty weightings.

Eq. (9) is solved iteratively, using, for example, a conjugate-gradient descent algorithm or projection onto convex sets, which can often lead to long reconstruction times making real-time applications of CS challenging.

**Fig. 7:**
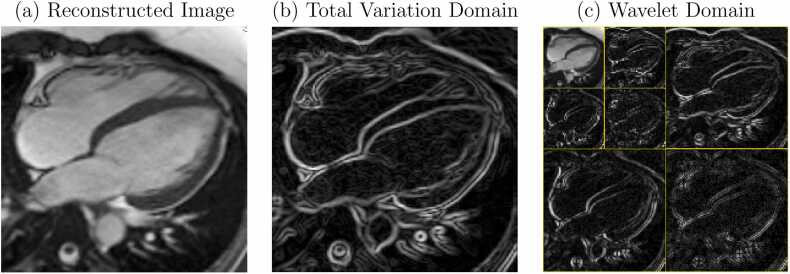
Sparse domains for CS reconstruction. The reconstructed MR image in (a) has sparse representations in the total variation (b) and wavelet (c) domains as only a small fraction of the total coefficients in these domains are non-zero. *CS* compressed sensing, *MR* magnetic resonance

Total variation (TV) and wavelet transformations are two examples of transforms often used in CS MRI reconstruction. These are depicted in Fig. 7, where the sparse representations of a reconstructed cardiac image in each of the domains are apparent. The TV is a first-order approximation of the spatial derivative; regions of relatively constant pixel value produce small values whereas tissue boundaries result in larger values. Minimizing the number of non-zero TV coefficients thus promotes large smooth regions in the image. The wavelet domain contains information about the presence of structure in the image at different scales, and is well known to provide a sparse representation of natural images, as is exemplified, for instance, in its use as the basis for JPEG 2000 image compression.

The k-t SPARSE [70] method, proposed for cardiac MRI, was the first application of CS for dynamic imaging. It utilizes random-order phase encoding in the k-t domain to ensure incoherent artifacts and incorporates sparsifying transforms in both the spatial and temporal dimensions. A wavelet transform, as previously discussed, was applied in the spatial dimensions. In the temporal dimension, a Fourier transform was applied, since it sparsifies smooth and periodic motion. In this manner, similarities between CS in the temporal dimension and UNFOLD, which applied filtering to the temporal frequency *f*, become apparent. Indeed, CS has also been applied to cardiac cine imaging with the x-f domain used directly as the sparse domain [71], and in that study it was shown to outperform k-t BLAST. Increased artifact incoherence can be achieved by replacing the random order but Cartesian phase encodes of k-t SPARSE with radial sampling. The iterative golden-angle radial sparse parallel MRI (iGRASP) technique [72], for instance, acquires radial spokes with golden angle (111.25^∘^) separation between successive spokes, ensuring relatively uniform k-space coverage for any arbitrary series of consecutive acquisitions.

The CS framework is also readily compatible with parallel imaging techniques; when **b** consists of multi-coil data and the encoding operator *E* incorporates coil sensitivity profiles, Eq. (9) is simply a regularized form of the iterative SENSE equation (Eq. (3)). As an example, the k-t SPARSE-SENSE approach, which utilized the sparsifying transforms from k-t SPARSE and a multi-coil array, was able to achieve eight-fold acceleration (*R* = 8) with comparable image quality to a two-fold accelerated GRAPPA reconstruction for first-pass cardiac perfusion [73]. The same approach applied to real-time cine, again with an acceleration factor of *R* = 8, was able to produce diagnostic images with a temporal resolution of 40–50 ms [74], [75].

As the potential of CS reconstruction for improving dynamic MRI became clear, more advanced methods incorporating motion estimation (ME) and motion correction (MC) were proposed [76], [77], [78], [79], [80], [81], [82]. One such method, k-t focal underdetermined system solver [76], [77], imposes sparsity not on a transform of the image itself but on the residual between the image and an initial image prediction. The prediction may be obtained using a RIGR-like method, or a more sophisticated ME/MC technique which estimates motion vector field between frames and applies these fields to fully sampled reference images.

The motion-adaptive spatio-temporal regularization [78] method similarly utilizes inter-frame motion, but without relying on a reference frame. Instead, forwards and backwards motion fields are estimated between every pair of adjacent frames from an initial reconstruction, and the sparsity constraint is applied to the difference between an estimated frame and its neighboring frame warped by the motion field to be in the same motion state. Motion can alternatively be estimated across groups of frames, reducing the impact artifacts in the initial reconstruction can have on motion fields in pairwise estimation [79], [80].

The CS framework is also applicable to non-real-time imaging applications where retrospective data sorting can be applied. The extra-dimensional iGRASP (XD-GRASP) [83] method arranges data in multiple temporal dimensions (depending on the application) and then applies different sparsity transforms along each. For instance, in an XD-GRASP reconstruction of 2D free-breathing cardiac imaging, the position in the cardiac cycle and the position in the respiratory cycle were used as the temporal dimensions [83]. The concept has also been applied for five-dimensional free-running cardiac imaging [84], [85] (three spatial dimensions, plus respiratory, and cardiac temporal dimensions) with a 3D golden-angle radial trajectory, where both the respiratory and cardiac signals are able to be extracted from the acquired k-space center, alleviating any requirement for a synchronized electrocardiogram signal [85].

An import consideration in the implementation of CS-based reconstructions is the choice of the penalty weighting(s) *λ*, as seen in Eq. (9). In general, no universally optimal value of *λ* exists, and the empirically determined values often used depend on multiple factors such as the acceleration factor, the sampling scheme, the image type, the data scaling, and the sparsifying transform [86]. Selecting a too-low value results in insufficient regularization, leading to an increased level of noise and artifacts in the image. Overly large values instead prioritize sparsity over data fidelity and tend to excessively smooth or blur the image in both the spatial and temporal dimensions [83]. More recently, techniques that optimize these weighting(s) over databases, as occurs in deep learning (discussed later), have been proposed [165].

**Fig. 8:**
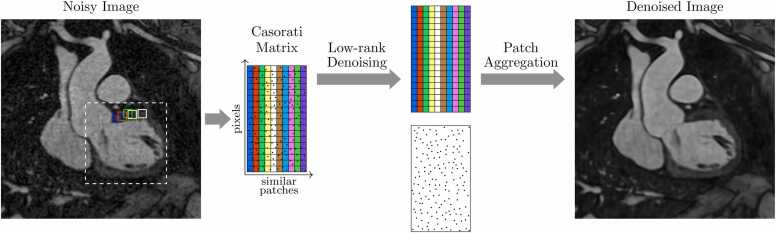
Low-rank patch-based denoising. Similar patches in the noisy image are collected and a Casorati matrix is formed from their pixel values. By reducing the rank of the matrix, incoherent noise is removed from the patches. Finally, the patches are aggregated to form a denoised image

Another class of methods closely related to CS are low-rank approaches [87], [88], [89], [90]. These are similarly constructed as a regularized minimization problem, but the sparsity penalty of CS is replaced with a penalty based on the rank of a Casorati matrix *M*, which is constructed from the dynamic image ***ρ*** in various ways (discussed below). The problem to solve becomes

(10) $$\arg\underset{\rho }{\min}∥E\rho -b{∥}_{2}^{2}+\lambda R\left(M\right),$$

where $R\left(M\right)$ is the rank of *M* [91]. Rank is a matrix property given by the number of linearly independent vectors that are required such that any column of the matrix can be constructed via a linear combination of the vectors. That is, if the matrix *M* consists of *K* columns and has rank 3, then there exist vectors **a**_1_, **a**_2_, and **a**_3_ such that every column **m**_*k*_ can be written as

(11) $$m_{k}=c_{1}a_{1}+c_{2}a_{2}+c_{2}a_{2},$$

a linear combination of the vectors. The idea is depicted in Fig. 8 where similar patches in the initial image are located and their pixels stacked in the Casorati matrix [92], [93], [94]. By applying a low-rank constraint to the matrix, incoherent noise is removed from the patch and aggregating the patches returns a denoised image.

Reconstruction methods that enforce the low rankness of Casorati matrices bear similarities to CS reconstruction, in that sparsity is enforced in the coefficient domain. That is, low-rank matrices require few non-zero coefficients *c*_1_, *c*_2_, etc. The key difference is that the sparsifying transformation is in this case dependent on the matrix (or image).

For dynamic imaging applications, low-rank methods can be used under the assumption that temporal variations can be well-described with only a few basis functions [91], [95], [96], [97]. In k-t sparsity and low-rank (SLR) [91], the matrix is arranged such that each row contained the time series for a given pixel, and a SVD is applied to enforce low rankness by retaining only the highest singular values. In k-t principal component analysis (PCA) [95], each column of *M* instead contains the temporal frequency profile of a given pixel, and a PCA is applied to the columns. Using only the first 10 principal components (enforcing $R\left(M\right)=10$), improvements can be seen over k-t BLAST.

Low-rank and sparse techniques have also been combined [82], [98], [99], [100]. For example, the *L* + *S* method [99] represents the high-dimensional image as the sum of a low-rank matrix *L*, which models the slowly varying background component, and a sparse matrix *S*, which captures the more-dynamic motion. This approach was able to provide high acceleration factors in a range of dynamic applications, including cardiac cine and cardiac perfusion.

## 7 Machine learning

In recent years, much attention has been focused on emerging machine-learning methods for image reconstruction. These approaches demonstrate promising potential for the next generation of reconstruction techniques, enabling increased acceleration factors, high reconstruction quality, and reduced computational time [101], [102], [103], [104], [105], [106].

The fundamental idea behind machine learning is that, with a sufficient amount of training data, correlations in the data can be iteratively learnt. For the deep-learning networks often employed in MRI, multiple layers exist between the input and output of the network, and parameters that determine how each layer is calculated from the previous one are learnt during the training stage. Since the prior information learnt by the network is derived from the data itself, rather than being a human-designed constraint imposed on the reconstruction as in CS, it is reasonable to expect deep learning may lead to improved reconstruction quality. Additionally, deep-learning networks have the advantage that, while the training stage may be slow and computationally expensive, the actual reconstruction stage is generally very fast. Nevertheless, optimal design of the network remains an open question.

As the technology matures, increased clinical adoption of deep-learning reconstruction is expected and present limitations will need to be addressed [107], [108]. These include the availability of large and unbiased datasets for training; the generalizability of methods beyond the scanner vendors, field strengths, and imaging sequences they were trained with; the interpretability of the networks, particularly important in establishing trust in the methods by clinicians; and the need for widespread clinical validation.

In this section, we consider proposed techniques as separated into three categories: supervised techniques, which rely on the existence of large databases of high-quality training data, self-supervised techniques, which do not require fully sampled training data, and subject-specific techniques, in which a distinct set of network parameters are learnt every time as part of the reconstruction process. Deep-learning-based image reconstruction is overviewed generally, with specific applications and extensions for the dynamic case highlighted.

### 7.1 Supervised techniques

Perhaps the most widely researched class of deep-learning techniques proposed for MRI reconstruction are supervised techniques, which rely on the existence of a fully sampled ground-truth image for every undersampled dataset used during the training stage, in order to calculate a loss between the known ground-truth and the deep-learning reconstruction. Alternatively, networks can be trained under a supervised regime to “mimic” much slower iterative methods such as CS by treating the reconstructions from those methods as the ground-truth objective.

One of the first MRI-reconstruction applications of supervised deep learning was a proposal to use a convolutional neural network (CNN) to obtain fully sampled images from the input of an undersampled image [101]. Here, convolutions with learnt kernels are applied between layers, and the network is trained using many fully sampled MR datasets that are retrospectively undersampled to provide zero-filled images. The network learns the implicit relationship between the zero-filled and fully sampled images, since the parameters are tuned iteratively to reduce the difference between the network output and the fully sampled images.

**Fig. 9:**
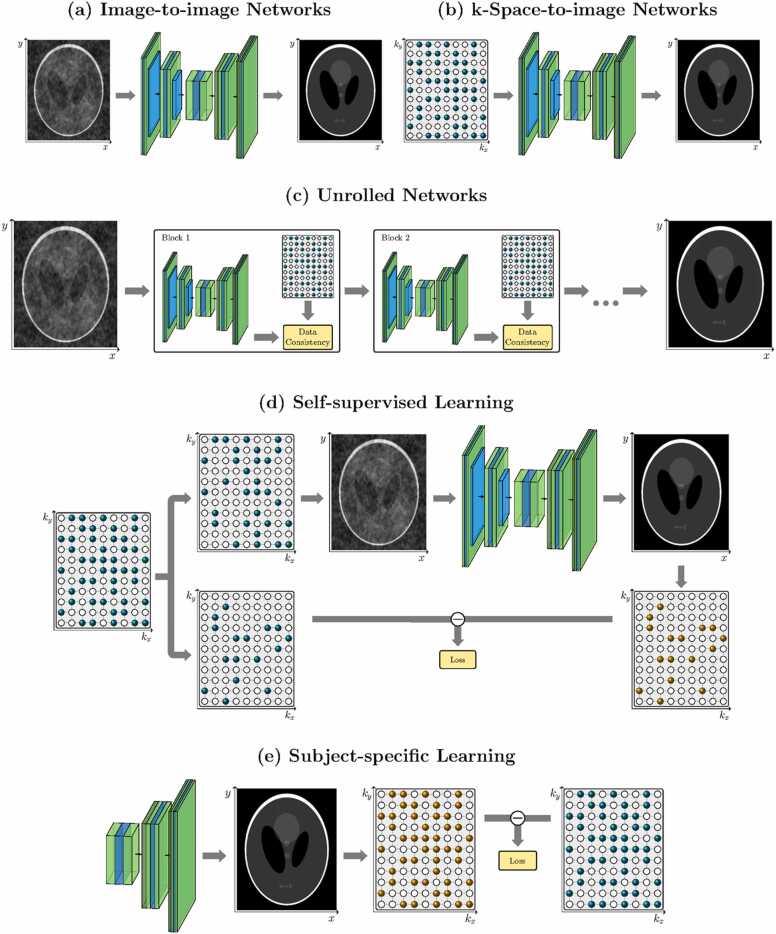
Deep-learning neural networks for image reconstruction. Network layers are formed of various operations, such as convolutions, non-linear activations, batch normalizations, max-pooling, and up-sampling. The input of the network can be in the image (a) or k-space (b) domains. Alternatively, networks may be embedded in unrolled iterative reconstructions (c). When fully sampled data are not available for training, networks may be trained from undersampled data in a self-supervised manner (d) or trained to determine network parameters unique to one specific image (e)

Many deep-learning MRI methods similarly tackle the problem as one of denoising a zero-filled image, as depicted in Fig. 9a, while others directly take the acquired k-space as an input (Fig. 9b) and thereby perform the dual roles of recovering missing data and performing an inverse Fourier transform [102], [109]. Alternatively, data consistency constraints may be imposed with an unrolled network architecture [110], [111], [112], [113], [114], as seen in Fig. 9c. In this scenario, the network effectively takes the place of the regularization term as seen in CS and low-rank formulations.

Various supervised techniques have been proposed specifically for dynamic imaging applications [111], [112], [113], [114], [115], [116], [117], [118], [119], [120], [121]. For example, a deep cascade of CNNs with data consistency constraints was proposed and applied to real-time 2D cardiac cine images [114]. The network was able to reconstruct a sequence of 30 frames, each 11-fold undersampled, within 10 s and outperformed existing state-of-the-art approaches including k-t SLR [91] and the *L* + *S* method [99]. Like UNFOLD, k-t SENSE and other methods that build on spatio-temporal sampling results, deep-learning networks are able to exploit the spatio-temporal correlations present in dynamic MR data. The deep cascade of CNNs is among several methods that utilize 3D (2D + time) convolutions [114], [115], [116], [117], [118]. Such spatio-temporal convolutions may be truly 3D, or can be constructed as a separable operation with a 2D spatial convolution followed by a 1D temporal convolution [122]. Separable convolutions decrease the number of trainable parameters to be learnt, allowing more efficient training [117], [122], with the potential cost of a reduction in the network’s ability to exploit correlations. In myocardial perfusion, it has been shown that true 3D spatio-temporal convolutions offer improved performance in the reduction of aliasing artifacts relative to separable convolutions [118]. Other examples of leveraging spatio-temporal correlations with deep-learning include a method combining 3D (2D + time) convolutions in x-t space with 3D (2D + temporal frequency) convolutions in x-f space [113], a method utilizing recurrent CNNs [119], and CINENet, which performs separable four-dimensional (4D) (3D + time) convolutions to enable 3D cine in a single breath-hold with a reconstruction time of just ∼ 5 s [120].

As was the case for CS methods, explicit ME and correction can be incorporated into deep-learning dynamic reconstruction techniques. Building on successful networks such as FlowNet [123] and recurrent all-pairs field transforms [124], which have been proposed for optical flow applications in computer vision, various networks have been proposed for ME in MRI [110], [111], [112], [125], [126], [127], [128], [129]. These networks take, as input, either a pair [126], [127], [128], [129] or group [111], [112], [125] of images in different motion states, and output non-rigid motion field(s) between these states. ME has been demonstrated for both respiratory [110], [128] and cardiac [111], [112], [125], [126] motion. By estimating both forwards and backwards motion fields, or estimating diffeomorphic fields which can be inverted [129], [130], the motion can be incorporated into the encoding operator of a data consistency term [131]. When incorporated as part of an unrolled iterative reconstruction, the ME network parameters can be learnt to optimize objectives based not only on the correctness of their estimated motion fields, but also on the quality of the final reconstructed images [110], [111], [112]. Recent work leveraging these concepts for cardiac cine was able to demonstrate excellent image quality from a 20-fold undersampled Cartesian acquisition [111].

### 7.2 Self-supervised techniques

The availability of training data of sufficient quality and quantity can pose a significant challenge to the feasibility of supervised techniques. This is particularly true in dynamic applications where it may be impossible to sample k-t space fully at the desired spatial and temporal resolution. To address this problem, various self-supervised methods, where training is implemented without fully sampled ground-truth images (or without ground-truth images paired with undersampled data), have been proposed [132], [133], [134], [135], [136], [137], [138], [139], [140], [141], [142].

The self-supervised learning via data undersampling (SSDU) [132] method, for instance, uses a training set comprised entirely of undersampled k-space acquisitions. The approach by which the framework is trained to recover high-quality images despite not being exposed to ground-truth images is depicted in Fig. 9d. Each undersampled k-space is separated into two disjoint subsets of k-space samples with a higher level of undersampling. An unrolled network incorporating data consistency is used to reconstruct images given the input of the zero-filled image corresponding to one subset of samples, with the loss calculated in k-space against the second subset.

The SSDU technique bears conceptual similarities with Noise2Noise [143], which was proposed in computer vision for denoising images and trained only with pairs of distinct noisy images of the same scene, and is one of a series of methods utilizing similar ideas for MRI reconstruction [133], [134], [135], [136], [137], [138], [139], [140], [144]. Among these is Phase2Phase [134], where the distinct undersampled k-spaces corresponded to data acquired in adjacent respiratory phases, DC-SiamNet [137], which utilizes contrastive learning to account for the overall image structure, and NLINV-Net [139] which jointly estimates coil sensitivity maps alongside the images. The latter was trained and applied to reconstruct real-time 2D cine images from ∼19-fold undersampled radial acquisitions, achieving comparable image quality to a CS implementation which could not be implemented online due to its computational demand [139]. SSDU itself has also been applied to dynamic CMR; a version utilizing multiple k-space mask pairs for each training sample [141] was applied to a simultaneous multi-slice acquisition of myocardial perfusion, with four-fold undersampling in each slice and a temporal resolution of 116 ms, where it was demonstrated to provided improved image quality and sharpness relative to CS-based techniques [142].

Generative and diffusion-based techniques have also been proposed for MRI and are trained to generate samples from a known image distribution [145], [146], [147], [148]. Since the training does not require paired samples of ground-truth and undersampled images, the high-quality images used in training need not exactly match the desired output. For instance, fully sampled static 2D images could be used in a framework proposed for cine reconstruction [146]. In another proposed generative-model-based technique, the network was trained using only undersampled data, and, in a similar manner to SSDU, the difference between a separate k-space acquisition and an undersampled k-space synthesized from the reconstruction was compared, here with a discriminator network [149]. While these techniques have yielded promising results for static MR applications, there has currently only been limited investigation into their use for dynamic reconstruction problems [150] and further research is warranted.

### 7.3 Subject-specific techniques

Subject-specific deep-learning methods form another class of reconstruction techniques [151], [152], [153], [154], [155], [156], [157], [158], [159], [160], [161], [162], [163]. These utilize deep neural networks while obviating the need for large amounts of training data. When applied to MRI, an unsupervised network can be trained to output an image (or series of images, for dynamic applications) given the input of either a latent variable [152], [153], [154] or image with random pixel values [155], [156], [157]. A forward model can then be applied to the output image, yielding corresponding k-space values which are compared against the acquired k-space to determine the network training loss, as is depicted in Fig. 9e. Since the network does not take specific information from the scan input, such as acquired k-space samples or a zero-filled image, and is not trained prior to reconstruction, a unique set of network parameters must be learnt for every scan. Thus, removing the requirement for large amounts of training data comes at the cost of long training/reconstruction times. The random-pixel input image case is known as the deep image prior approach [164] and relies on a CNN architecture. It has been successfully applied to stationary cardiac magnetic resonance fingerprinting (MRF) [155] as well as 2D cine MRF [156], [157]. Its success lie in the fact that the CNN architecture is able to learn natural image structure faster than it learns noise, and provided the network training is stopped at an appropriate time an image that well-fits the acquired k-space measurements but has not been over-fitted to noise may be obtained. When the technique was adapted for dynamic cine imaging [156], two networks were utilized in a manner that allowed temporal correlations to be exploited. The first generated a spatial basis consisting of a series of images and the second generated a temporal basis that determined the weights for combining the spatial basis images at each time point.

The SSDU method has also been adapted for subject-specific reconstruction in the scenario that a training database of undersampled scans is not available [158]. In this case, the acquired undersampled data are split into three disjoint subsets, two for input and loss-calculation, as before, with the third deployed for self-validation, to prevent over-fitting. Applied to real-time cine [159], the subject-specific SSDU was able to achieve good quality images in cases where the spatio-temporal correlations were not effectively learned in database-trained SSDU.

Another technique for achieving subject-specific deep-learning-based reconstruction involves utilizing implicit neural representations (INRs) [160], [161], [162], [163]. INRs use a neural network, typically a fully connected multi-layer perceptron, to represent the image as a continuous function of the image-space coordinates *x*, *y* and, for 3D images, *z*. Again, these networks must be trained for every image or image series being reconstructed. At inference, each coordinate corresponding to a pixel position is passed to the network, and the network outputs the value of that pixel. In this way, INRs can also be employed to perform implicit super resolution, since the resolution of the pixel grid can be set arbitrarily. In dynamic cardiac MR, INRs have been proposed for 2D [160], [161] and 3D [162] cine. Additionally, a k-space-based INR has been proposed [163], where k-space values corresponding to a particular coil and time-point, rather than pixel values, were produced by the network. The technique was able to achieve 30-frame cine images with 2 mm × 2 mm resolution from a single heartbeat with good image quality, significantly outperforming CS- and low-rank-plus-sparse-based reconstructions.

## 8 Conclusion

The field of dynamic cardiac MR image reconstruction has advanced greatly over the past three decades and remains today an active area of research. From its early days when low-resolution 2D dynamic images were only possible at low temporal resolution, now sophisticated reconstruction techniques exploiting parallel imaging, spatio-temporal redundancies, prior information, and the latest machine learning architectures are able to provide good-quality high-resolution images at high temporal resolution. Without such innovations, the role of modern MRI in dynamic imaging applications such as cardiac cine, speech MRI, and perfusion would not be possible.

## Funding

The authors acknowledge financial support from (1) King’s BHF Centre for Award Excellence PG/18/59/33955 and RG/20/1/34802, (2) 10.13039/501100000266EPSRC EP/V044087/1 (3) Wellcome EPSRC Centre for Medical Engineering (NS/A000049/1), (4) Millennium Institute for Intelligent Healthcare Engineering ICN2021_004, 10.13039/501100002850FONDECYT 1210637 and 1210638, (5) IMPACT, Center of Interventional Medicine for Precision and Advanced Cellular Therapy, Santiago, Chile. ANID-Basal funding for Scientific and Technological Center of Excellence, IMPACT, #FB210024 (6) the Department of Health through the 10.13039/501100000272National Institute for Health Research (NIHR) comprehensive Biomedical Research Centre award, (7) NIHR Cardiovascular MedTech Co-operative, (8) the 10.13039/501100005713Technical University of Munich - Institute for Advanced Study and (9) the Government of Denmark. The views expressed are those of the authors and not necessarily those of the BHF, NHS, the NIHR or the Department of Health.

## Author contributions

**René M. Botnar:** Writing – review & editing, Conceptualization. **Claudia Prieto:** Writing – review & editing, Conceptualization. **Andrew Phair:** Writing – review & editing, Writing – original draft, Conceptualization.

## Declaration of competing interests

The authors declare that they have no known competing financial interests or personal relationships that could have appeared to influence the work reported in this paper.

## Appendix A Appendix

### A.1 The Fourier transform

Mathematically, the k-space *b*(**k**) is related to the image $\rho \left(x\right)$ by

(A.1) $$b\left(k\right)=\int \rho \left(x\right)e^{-ik\cdot x}\;dx,$$

which we recognize as the continuous Fourier transform in two or three dimensions, depending on the number of k-space and image-space variables in the vectors **k** and **x**, respectively. The image can thus be recovered from the acquired k-space data by applying the inverse Fourier transform,

(A.2) $$\rho \left(x\right)=\frac{1}{{\left(2\pi \right)}^{Q}}\int b\left(k\right)e^{ik\cdot x}\;dk,$$

where *Q* is the number of dimensions. In practice, both the acquired k-space data and the image being reconstructed consist of discrete data samples, and so the discrete inverse Fourier transform is instead considered. For 2D imaging, this may be written as

(A.3) $$\rho_{u,v}=\frac{1}{N}\frac{1}{M}\sum_{n=1}^{N}\sum_{m=1}^{M}b_{n,m}e^{i\left(x_{u}{\left(k_{x}\right)}_{n}+y_{v}{\left(k_{y}\right)}_{m}\right)},$$

which can also be generalized to 3D.

### A.2 SENSE equations

In SENSE, a set of linear equations may be written for each pixel in the reduced FOV describing how the aliased pixels are formed as the superposition of multiple coil-weighted pixels. In general, these take the form

(A.4) $$\begin{array}{cc} a_{1}=s_{1,1}\rho_{1}+s_{1,2} & \rho_{2}+\cdots +s_{1,R}\rho_{R}\\ a_{2}=s_{2,1}\rho_{1}+s_{2,2} & \rho_{2}+\cdots +s_{2,R}\rho_{R}\\ & \vdots \\ a_{N_{c}}=s_{N_{c},1}\rho_{1}+s_{N_{c},2} & \rho_{2}+\cdots +s_{N_{c},R}\rho_{R} \end{array}$$

and in the particular case illustrated in Fig. 4, where *N*_*c*_ = 2 and *R* = 2, they become

(A.5) $$\begin{array}{ccc} a_{1} & = & s_{1,1}\rho_{1}+s_{1,2}\rho_{2}\\ a_{2} & = & s_{2,1}\rho_{1}+s_{2,2}\rho_{2}. \end{array}$$

### A.3 SMASH k-space synthesis

In SMASH, k-space line measurements acquired in different coils are linearly combined with weights chosen such that the combined coil sensitivity profile closely matches a spatial harmonic which modulates the exponential term in the integrand of the Fourier transform (Eq. (A.1)). Expressing the linear combination in terms of the Fourier transform definition of k-space gives

(A.6) $$\sum_{j=1}^{N_{c}}g_{j}b_{j}\left(k_{x},k_{y}\right)=\iint \sum_{j=1}^{N_{c}}g_{j}S_{j}\left(x,y\right)\rho \left(x,y\right)e^{-i\left(k_{x}x+k_{y}y\right)}\;dx\;dy$$

where *g*_*j*_ are the weights. If the weights are chosen such that

(A.7) $$\sum_{j=1}^{N_{c}}g_{j}S_{j}\left(x,y\right)=e^{im\Delta k_{y}y}$$

then the combination becomes

(A.8) $$\begin{array}{ccc} \overset{N_{c}}{\underset{j=1}{\sum }}g_{j}b_{j}\left(k_{x},k_{y}\right) & = & \iint \rho \left(x,y\right)e^{-i\left(k_{x}x+\left(k_{y}-m\Delta k_{y}\right)y\right)}\;dx\;dy\\ & = & b\left(k_{x},k_{y}-m\Delta k_{y}\right), \end{array}$$

which is the k-space shifted by *m* grid units in the *k*_*y*_ direction. Therefore, applying the linear combination to an acquired line in k-space yields a neighboring, unacquired line.

### A.4 GRAPPA k-space synthesis

In GRAPPA, all acquired samples that sit within a k-space neighborhood of an unacquired sample contribute to its synthesis via a linear combination which utilizes a calibrated kernel of weights. This may be expressed as

(A.9) $$b_{\gamma }\left(s,t\right)=\sum_{j}\sum_{u}\sum_{v}g_{u,v,\gamma ,j}b_{j}\left(s+u\Delta k_{x},t+v\Delta k_{y}\right),$$

where $b_{\gamma }\left(s,t\right)$ is the k-space value at the location (*k*_*x*_, *k*_*y*_) = (*s*, *t*) in the *γ*th coil, *g*_*u*,*v*,*γ*,*j*_ is the kernel element (linear combination weight) used for the synthesis of the center sample in the *γ*th coil from the sample in the (*u*, *v*) kernel position in the *j*th coil, and the second and third summations are over all values of *u* and *v* that correspond to acquired samples in the neighborhood.

The applicability of weights calibrated in the ACS region to the rest of k-space can be demonstrated by considering Eq. (A.9) with the Fourier transform definition for the k-space samples (Eq. (A.1)) substituted in,

(A.10) $$\iint \rho_{\gamma }\left(x,y\right)e^{-i\left(sx+ty\right)}\;dx\;dy=\iint \sum_{j}\sum_{u}\sum_{v}g_{u,v,\gamma ,j}\rho_{j}\left(x,y\right)e^{-i\left(\left(s+u\Delta k_{x}\right)x+\left(t+v\Delta k_{y}\right)y\right)}\;dx\;dy.$$

Since the kernel elements *g*_*u*,*v*,*γ*,*j*_ have been determined over the ACS region, rather than at a single position, we can assume this relationship is not a coincidental equality of integrals but instead due to the equality of the underlying integrands, as in the composite sensitivity profile which takes the place of Fourier basis functions in SMASH. Equating the integrands and multiplying both sides by $\exp\left(i\left(U\Delta k_{x}+V\Delta k_{y}\right)\right)$, for arbitrary integers *U* and *V*, yields

(A.11) $$\rho_{\gamma }\left(x,y\right)e^{-i\left(\left(s+U\Delta k_{x}\right)x+\left(t+V\Delta k_{y}\right)y\right)}=\sum_{j}\sum_{u}\sum_{v}g_{u,v,\gamma ,j}\rho_{j}\left(x,y\right)e^{-i\left(\left(s+\left(u+U\right)\Delta k_{x}\right)x+\left(t+\left(v+V\right)\Delta k_{y}\right)y\right)}.$$

Integrating both sides of Eq. (A.11) with respect to *x* and *y* gives

(A.12) $$b_{\gamma }\left(s+U\Delta k_{x},t+V\Delta k_{y}\right)=\sum_{j}\sum_{u}\sum_{v}g_{u,v,\gamma ,j}b_{j}\left(s+\left(u+U\right)\Delta k_{x},t+\left(v+V\right)\Delta k_{y}\right),$$

which describes, as required, the synthesis of a sample at the shifted location $\left(k_{x},k_{y}\right)=\left(s+U\Delta k_{x},t+V\Delta k_{y}\right)$ using the neighboring samples in the same relative positions and the same kernel of weights.

### A.5 k-t BLAST and k-t SENSE equations

For a k-t BLAST acquisition, if a total of *H* points *β* in x-f space alias to the same position *α*, these points must satisfy

(A.13) $$\alpha =\beta_{1}+\beta_{2}+\cdots +\beta_{H},$$

or, in the multi-coil case where there is an x-f space corresponding to each coil and the coil sensitivity profiles are assumed to be time-invariant,

(A.14) $$\begin{array}{cc} \alpha_{1}=s_{1,1}\beta_{1}+s_{1,2} & \beta_{2}+\cdots +s_{1,H}\beta_{H}\\ \alpha_{2}=s_{2,1}\beta_{1}+s_{2,2} & \beta_{2}+\cdots +s_{2,H}\beta_{H}\\ & \vdots \\ \alpha_{N_{c}}=s_{N_{c},1}\beta_{1}+s_{N_{c},2} & \beta_{2}+\cdots +s_{N_{c},H}\beta_{H}. \end{array}$$
